# Stress distribution in different tooth-implant supported mandibular prostheses rehabilitating unilateral free-end saddle: a biomechanical comparative finite element study

**DOI:** 10.1186/s12903-026-09173-x

**Published:** 2026-07-22

**Authors:** Haitham Amr Mohammed, Maha Nagy Mohamed Kamal, Abdallah Shokry

**Affiliations:** 1 Fixed Prosthodontics Department, Faculty of dentistry, Fayoum University, Al Fayyum, Egypt; 2 Removable Prosthodontics Department, Faculty of dentistry, British University in Egypt (BUE), Elshrouk, Cairo Governorate Egypt; 3 Mechanical Engineering Department, Faculty of Engineering, Fayoum University, Al Fayyum, Egypt

**Keywords:** Double OT, Unilateral PD, Finite element analysis, Ttooth-implant supported fixed bridge, Extra-coronal attachment, OT vertical attachment

## Abstract

**Aim of the study:**

Utilizing Finite Element Analysis, this research biomechanically assessed three prosthetic designs for mandibular unilateral free-end saddle, specifically interrogating the magnitude and distribution of stresses transmitted to the terminal anterior abutment and the supporting posterior implant.

**Materials and methods:**

A mandibular unilateral free-end saddle educational cast, with the first premolar as the last standing abutment tooth, was used. The abutment teeth of the free end saddle side were prepared to accommodate two splinted crowns. Then the cast was scanned, and an implant analogue was digitally inserted at the second molar area using Blender4dental software. Three different treatment modalities were digitally designed and collected using Blender4dental software, then grouped as follows: Group 1: Tooth-Implant supported fixed-fixed bridge. Group 2: OT vertical attachment retained removable bridge. Group 3: Double OT cap retained removable partial denture. The preparatory stage involved editing the STL file within the Meshmixer software environment. Subsequent finite element analysis FEA was performed using Abaqus, where specific occlusal loads were applied over the area to be examined: 200 N (vertical) and 23.5 N (tangential) on the molars, and 140 N (vertical) and 16.45 N (tangential) on the canines and premolars. The von Misses stress levels induced around the anterior abutments, at the residual ridge area, and around the implant were measured and compared.

**Results:**

Regarding the stresses exerted by the whole design, it was found that, the lowest stresses were found with design 3 (Double OT cap retained removable partial denture), while the highest stresses were found with design 2 (OT vertical attachment retained removable bridge). Regarding von-Mises stresses on the prepared abutments and the residual ridge, the lowest von Mises stresses were found in design 3, while the highest von Mises stresses were found in design 1 (Tooth-Implant supported fixed-fixed bridge). Regarding von-Mises stresses on the implant, the lowest Von Mises stresses were found in design 3, while the highest Von Mises stresses were found in design 2.

**Conclusion:**

RPD restored by double OT and ball and socket attachment exerted the least stress on the abutment, the residual ridge, and the implant; this design is recommended to be used with weak abutments, flat ridge, and\ or with imperfect implant stability. The fixed bridge design showed the highest stresses exerted on the anterior abutments and the residual ridge due to the absence of resiliency between the crowns and the teeth. This design could be indicated with strong abutments and well-formed ridges. OT vertical attachment retained removable bridge design showed the highest stresses exerted on the posterior implant, carrying a slight torquing force, resulting in more load concentration on the implant compared to the other two designs.

## Background

Rehabilitating a lower unilateral distal extension saddle (Kennedy Class II) is a long-standing challenge in dentistry due to the absence of a distal abutment. Conventional options like removable partial dentures (RPDs) are non-invasive and cost-effective, they often suffer from poor stability, bulkiness, and potential abutment damage, while cantilevered fixed partial dentures risk overloading the terminal abutment and places significant biomechanical stress on the prosthesis and remaining teeth. To address these issues, attachment-retained prostheses offer a superior alternative; by fully covering the abutments, they minimize caries risk compared to clasp assemblies, and their stress-breaking properties enhance stability and biomechanics, allowing for effective unilateral RPD designs [1–3]. 

The clinical scope of the present study is to offer alternative treatment options for lower Kennedy Class II cases when the traditional treatment option (Vitallium partial denture) is contraindicated for many reasons, including the inability to obtain cross-arch stabilization, patient rejection of metal clasp display with preference for more fixed restorations, or cases requiring additional support, better retention, and effective occlusion from the free-end saddle area through strategic distal implant placement [4, 5]. As documented in the recent prosthodontic literature, implant-supported fixed restorations remain the first-line treatment when clinical conditions permit, offering superior stability and chewing efficiency [6, 7]. However, in selected circumstances, combining teeth with implants serves as a valuable second-line strategy when the number, condition, or distribution of remaining natural teeth is insufficient to support traditional fixed prostheses due to anatomical limitations, the need to maintain proprioception, budgetary constraints, or patient preference [4, 8, 9]. In challenging clinical situations, such as following tumour surgery, bone resection, or in patients with limited remaining abutment teeth, connecting implants to teeth or utilizing implant-assisted removable partial dentures can help support neighbouring natural teeth and restore both function and appearance while reducing financial and surgical burdens [7, 10].

Based on the available evidences, tooth-implant supported prosthesis ( TISP) are specific and should be indicated for situations where conventional treatment alternatives are either contraindicated or impractical as in (1) posterior mandibular or maxillary regions where anatomical limitations (e.g., proximity to the inferior alveolar nerve, maxillary sinus, or narrow alveolar ridge) preclude placement of the ideal number of implants [4, 11]; (2) cases where the remaining natural tooth exhibits favorable periodontal and endodontic health, with sufficient periodontal ligament surface area to accommodate the additional occlusal load transfer [11, 12]; (3) financial or anatomical constraints that limit the patient to a single implant rather than multiple implants for distal extension situations [3]; and (4) patients who refuse removable prostheses but present with bone quality or quantity insufficient for a fully implant-supported fixed restoration [13]. In contrast, tooth-implant connection is contraindicated in patients with active periodontitis, compromised endodontic status of the abutment tooth, poor oral hygiene compliance, or when multiple implants can be predictably placed without additional morbidity [12, 14].

When properly executed, joining teeth and implants offers a treatment option with reduced cost while eliminating the need for extensive removable appliances, significantly enhancing patient comfort and perceived quality of life in carefully selected cases [7, 9]. The use of a solitary dental implant placed in the distal edentulous area to serve as an abutment for a fixed prosthesis effectively converts a precarious Kennedy Class II situation into a more biomechanically stable configuration [15, 16].

Despite its clinical application, the biomechanical rationale of connecting a mobile tooth to a rigid implant remains a contentious topic in prosthodontic literature. The fundamental concern lies in the mismatch of the support mechanisms: a natural tooth possesses physiological mobility afforded by the periodontal ligament (PDL), while an implant is directly anchored to bone with virtually no resilience [17]. This discrepancy can lead to differential vertical settlement under load.

Natural teeth exhibit physiologic mobility of approximately 28 to 100 μm, mediated by the viscoelastic properties of the periodontal ligament (PDL), whereas osseointegrated implants demonstrate negligible mobility (3–5 μm) due to direct bone-implant contact [18, 19]. This differential mobility under functional loading creates a mechanical conflict when rigidly connected: the implant acts as a fulcrum, potentially leading to intrusive forces on the natural tooth, a phenomenon termed “tooth intrusion” or “implant-assisted tooth intrusion” [20, 21]. The clinical significance of this biomechanical mismatch is underscored by reports of progressive intrusion ranging from 0.2 to 4.0 mm over follow-up periods, which may compromise occlusal stability, esthetics, and ultimately the prognosis of the natural tooth abutment [22, 23].

This biomechanical incompatibility is hypothesized to be a source of significant complications. Stress concentrations may arise at the implant-bone interface, potentially leading to marginal bone loss or, in extreme cases, implant failure. Furthermore, the rigid connection may transfer non-axial forces to the natural abutment tooth, risking pulpal, periodontal, or restorative damage, such as cement failure or screw loosening [24].

The longevity of a (TISP) is critically dependent on the health of both the implant and the connected natural tooth. The “splinting effect” intended to protect the tooth may instead subject it to abnormal forces, potentially accelerating its loss and, paradoxically, compromising the overall prosthetic outcome [25]. This risk necessitates careful patient selection and a thorough understanding of the biomechanics involved.

In light of these concerns, researchers have sought to quantify and visualize the stress distribution in (TISPs) using advanced engineering tools. The Finite Element Analysis (FEA) method has become an indispensable virtual tool in dental biomechanics, allowing for the detailed simulation of complex structures and the prediction of stress patterns under various loading conditions [26].

Recent FEA studies have consistently highlighted the critical role of the connector between the tooth and implant. Rigid connectors have been shown to generate high stress levels within the abutment’s periodontal ligaments and the cortical bone around the implant neck, which are the most vulnerable regions [27]. This underscores the potential risks of a direct, non-resilient connection.

As an alternative to rigid fixation, non-rigid connectors or stress-breaking elements have been proposed to mitigate the ill effects of the support mismatch. These designs aim to allow for the independent micro-movement of the natural tooth, thereby dissipating stress and protecting both the implant and the tooth [28]. Consequently, contemporary management strategies emphasize the use of stress-breaking mechanisms, such as non-rigid connectors positioned on the implant side, to accommodate differential mobility and reduce strain transmission to the tooth-PDL complex [14, 29]. However, their clinical efficacy and potential to introduce other complications, such as food impaction or connector failure, remain subjects of investigation.

The choice of prosthesis design extends beyond the type of connection. The configuration of the prosthesis itself, such as the number and position of pontics, the cantilever length, and the occlusal scheme, are all critical determinants of the resulting biomechanical environment [30]. Optimizing these parameters is essential for the long-term success of the restoration.

The material composition of the prosthetic superstructure is another pivotal factor influencing stress distribution. The use of high-strength zirconia frameworks versus traditional metal-ceramic systems can alter how masticatory loads are transferred to the underlying supporting structures due to differences in their elastic modulus. The clinical performance of (TISPs) has been evaluated in longitudinal studies and it was reported that while (TISPs) exhibit acceptable survival rates, they are associated with a higher incidence of technical complications, such as porcelain fracture and connector issues, compared to solely implant-supported prostheses [31]. Several recent FEA studies have modelled the mandibular unilateral distal extension scenario. For instance, a 2021 study by El-Anwar et al. concluded that a non-rigid connection significantly reduced stress in the periodontal ligament and peri-implant bone compared to a rigid connection, suggesting a potential biomechanical advantage [29].

Conversely, a different FEA investigation by Ueda et al. [27], reported that while non-rigid connectors reduced stress on the tooth, they could simultaneously increase the stress magnitude on the distal implant components, particularly under oblique loading conditions [28]. This highlights the complex and often counterintuitive nature of stress distribution in these systems.

When comparing prosthetic options, a finite element study by Bacchi et al. evaluated different designs for a lower free-end saddle and revealed that an implant-supported prosthesis with a distal implant generally produced the most favourable and homogeneous stress pattern in the supporting bone [30].

Despite the valuable insights from previous research, a direct comparative FEA of multiple (TISP) designs—considering variations in connection type, framework material, and pontic configuration under standardized loading conditions—is not fully explored in the recent literature. Many studies focus on a single variable, leaving a gap for a comprehensive analysis [27].

Furthermore, the specific biomechanical response of the mandibular bone, with its distinct cortical and cancellous structure, to the various (TISP) designs used in unilateral cases warrants more detailed investigation, the direction of occlusal loads for example, add layers of complexity that must be accurately modelled [32].

The current study’s clinical goal was to provide lower Kennedy class II patients with alternative treatment options when the conventional options as Vitallium partial denture or cantilever fixed bridge are not indicated, in addition to, assessing the stresses exerted by several mandibular tooth-implant-supported prosthesis designs on the supporting abutments, ridge, and implants used for the rehabilitation of a unilateral free-end saddle using 3D FEA. The investigation will simulate realistic masticatory loading to provide clinically relevant data.

The null hypothesis of this study predicts no significant difference in the magnitude and distribution of von Mises stress within the abutment teeth, the implant, and the residual bone structures among the different prosthetic designs evaluated. It is anticipated that the results will offer clinicians useful evidence-based recommendations help in selecting the most biomechanically sound prosthetic design for this challenging clinical situation.

## Materials and methods

“In accordance with the Declaration of Helsinki.” This study was approved by the Ethical Committee of the British University in Egypt (BUE) with research approval number 25–02.

*Procedures of model making*: an educational cast of mandibular Kennedy class II, having the first premolar as the last standing abutment, was used. First, the canine and the first premolar of the edentulous side were prepared with 1 mm deep chamfer finish line to receive two splinted porcelain veneered metal crowns. Then the prepared cast was scanned using a desktop scanner (3Shape E2 with 1920 × 1080| 1920 × 1200 resolution). The produced standard tessellation language (STL) file was manipulated using Blender4dental software, and an implant analogue was digitally inserted at the second molar area using Blender4dental software. Implant alignment was adjusted to be parallel to the splinted crowns path of insertion anteriorly. Three different treatment modalities supported and retained anteriorly by the canine and the 1st premolar abutments and posteriorly by the implant were digitally designed, assembled using Blender4dental software, and grouped as follows:

- Group 1: Tooth-Implant supported fixed-fixed bridge.

**Fig. 1:**
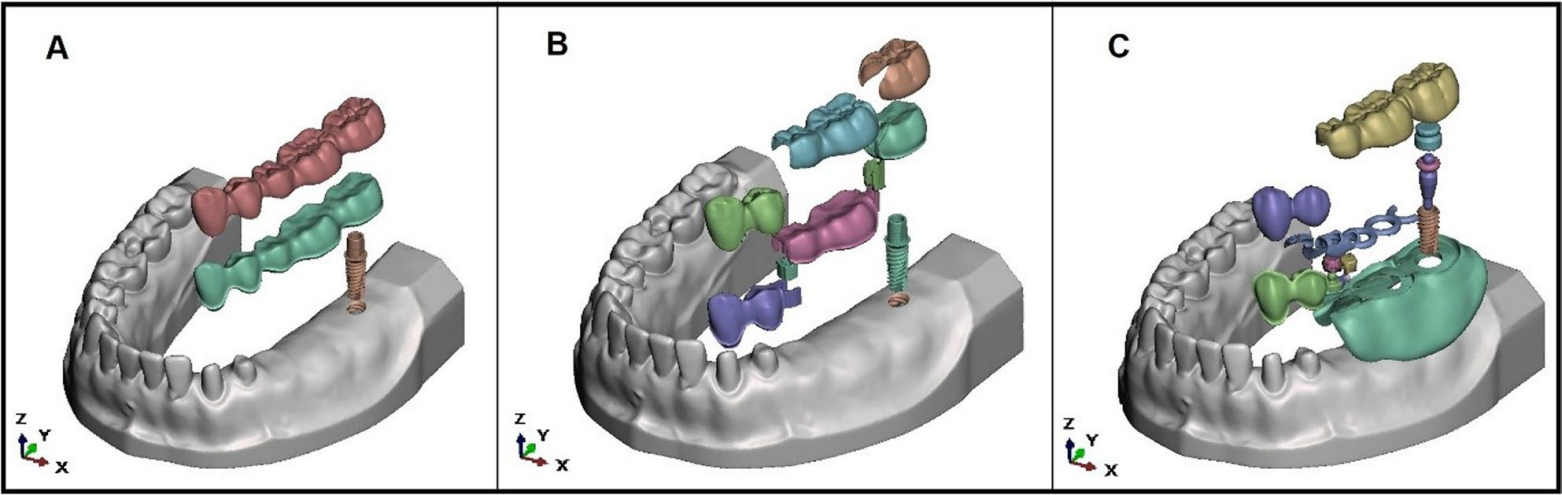
Three different treatment modalities for mandibular unilateral free-end saddle. **A**: Tooth-Implant supported fixed-fixed bridge (green color represents metal part of the bridge, red color represents the porcelain part of the bridge. **B**: OT vertical attachment retained removable bridge. (Purple &green crowns represents porcelain fused to metal -PFM- splinted crowns, pink & blue crowns represent the pontic part of the restoration, dark green & brown crowns represent the PFM crown over the implant) **C**: Double OT cap retained removable partial denture. (Purple &green crowns represents porcelain fused to metal -PFM- splinted crowns, bule color represents the denture base, purple meshwork represents the metal framework part of the RPD, Blue color represents the ball attachment housing, and the brown color represents the implant & PD teeth)

Digitally designed five-unit PFM fixed bridge extended from the canine to the second molar splinting the anterior abutments “canine and first premolar” with the posterior implant replacing the second molar (Fig. 1A).

- Group 2: OT vertical attachment retained removable bridge.

A removable bridge was constructed with an OT vertical attachment. Anteriorly, the attachment was used as an extra-coronal attachment projected from the splinted crowns of the canine and the 1st premolar, while posteriorly, the attachment was used as an intra-coronal attachment inserted inside the crown covering the implant (Fig. 1B).

- Group 3: Double OT cap retained RPD.

A unilateral RPD was retained with a double OT cap attached to the splinted crowns on the canine and 1st premolar anteriorly, and with a ball & socket attachment used on the implant posteriorly (Fig. 1C).

*Finite element analysis (FEA)*: was performed to evaluate the stresses transmitted from the different prosthesis designs to the supporting abutments (canine &1st premolar) anteriorly, the edentulous ridge, and the implant posteriorly when occlusal-like forces were applied.

The current investigations commenced with the digital sculpting and refinement of three distinct denture designs. The process initiated with their foundational STL files, which were imported into Meshmixer software. Here, the raw geometric data was meticulously curated; each model was transformed into a robust solid part, a critical step that eliminates any digital imperfections and ensures a perfectly watertight, analysable structure. Furthermore, strategic volumetric enhancements were applied to key regions, fortifying the digital models to accurately represent their physical counterparts and meet the stringent demands of subsequent engineering simulation.

**Fig. 2:**
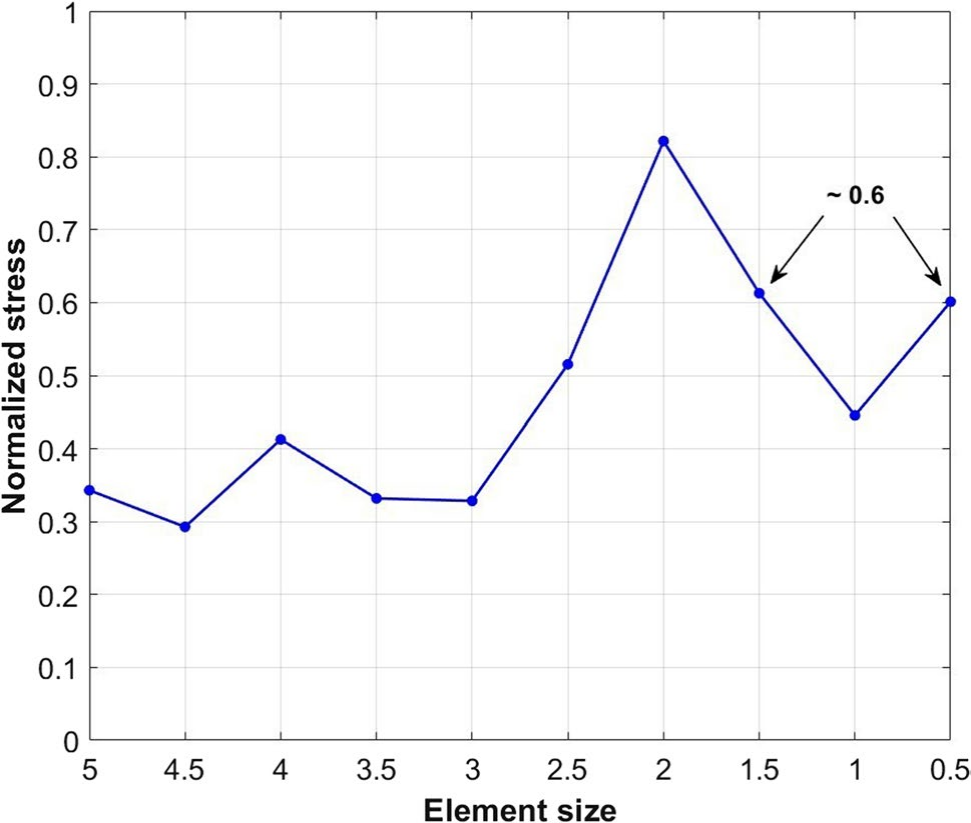
Normalized von Mises stress as a function of element size, highlighting the mesh-dependent response of the mandibular model

The prepared solid models were then transitioned into the Abaqus simulation environment for advanced finite element analysis (FEA). The core of this phase was discretization, a process where the continuous geometry was subdivided into a finite element mesh. As the mandible geometry was imported via STL files, its complex, free-form surfaces could not be easily partitioned for structured hexahedral meshing. A quantitative convergence analysis for the mandibular region charted the relationship between element size, ranging from 5 mm down to 0.5 mm, and the resultant normalized von Mises stress, as shown in (Fig. 2).

The meshing strategy used scalable fidelity, tailoring element size to each component’s importance. Ultra-fine 0.2 mm resolution was applied to tiny features like the plastic cap in design 3 (creating over 116,000 elements), while the larger mandible used a practical 1.5 mm size. This hierarchical approach optimized computational resources by maximizing detail on critical small features without sacrificing accuracy on larger structures. As a result, total element counts ranged from 1.1 to 2.0 million (node counts 1.7–3.0 million), directly reflecting the designs’ geometric complexity. This significant mesh investment was essential for generating precise stress fields, providing a high-resolution understanding of each denture design’s mechanical behavior and performance limits under operational loads.

Materials characteristics [33] of the 3D model are summarized in (Table 1). The materials are modelled as homogeneous, isotropic, and exhibiting linear elastic response.

**Table 1:** Mechanical properties of the materials used

Element	Material	Poisson’s ratio (ν)	Modulus of elasticity (GPa)	Density (g/cm³)
Cortical bone 1.2 mm outer layer of the jawbone	Bone	0.3	13.7	1.1
Trabecular bone III inner part of the jawbone	Bone	0.3	1,6	0.5
Porcelain facing	ceramic	0.19	65	2.1–2.6
Metal crowns	Nickel-chromium alloy	0.26 to 0.34	190 to 220	7.750 to 8.650
Attachment	Cobalt chromium alloy	0.29–0.33	190–253	8.4–8.8
Implant	Titanium (TiAl6V4)	0.31–0.34	114	4.43
Attachment & Metal housing & metal framework	Cobalt chromium alloy	0.29–0.33	190–253	8.4–8.8
Rubber cap	Silicon	0.48	0.05 Gpa	1.3
Acrylic teeth & acrylic denture base	PMMA	0.3	22.55 Gpa	1.188

In the interest of a controlled comparative analysis, a simplified boundary condition was strategically implemented, replacing a physiologically accurate temporomandibular joint model with a fixed constraint at the distal mandibular cut plane. While this abstraction does not capture the full complexity of natural joint dynamics, it was uniformly applied across all design variants. This methodological consistency ensures that any divergence in mechanical performance - stress distribution or displacement - can be attributed unequivocally to differences in design geometry rather than to variations in constraint behaviour. Consequently, this approach safeguards the validity of the relative performance assessment, providing a reliable foundation for design selection even within a simplified mechanical environment. To establish the biomechanical context of the simulation, a dual approach to boundary conditions was implemented. The mandible was modelled with fully fixed constraints at its bilateral ends, creating a stable foundational base by restricting all translational and rotational degrees of freedom. On the edentulous side, physiologically informed masticatory loads were applied to key occlusal surfaces. A higher force magnitude of 200 N vertically and 23.5 N tangentially was imposed on the first and second molars to simulate intense grinding forces, while a reduced yet significant load of 140 N vertical and 16.45 N tangential force was applied to the premolars, reflecting the gradient of force distribution along the jaw. These values, referenced from established [34], ensure that the loading regime is accurately represented in vivo functional conditions.

## Results

**Fig. 3:**
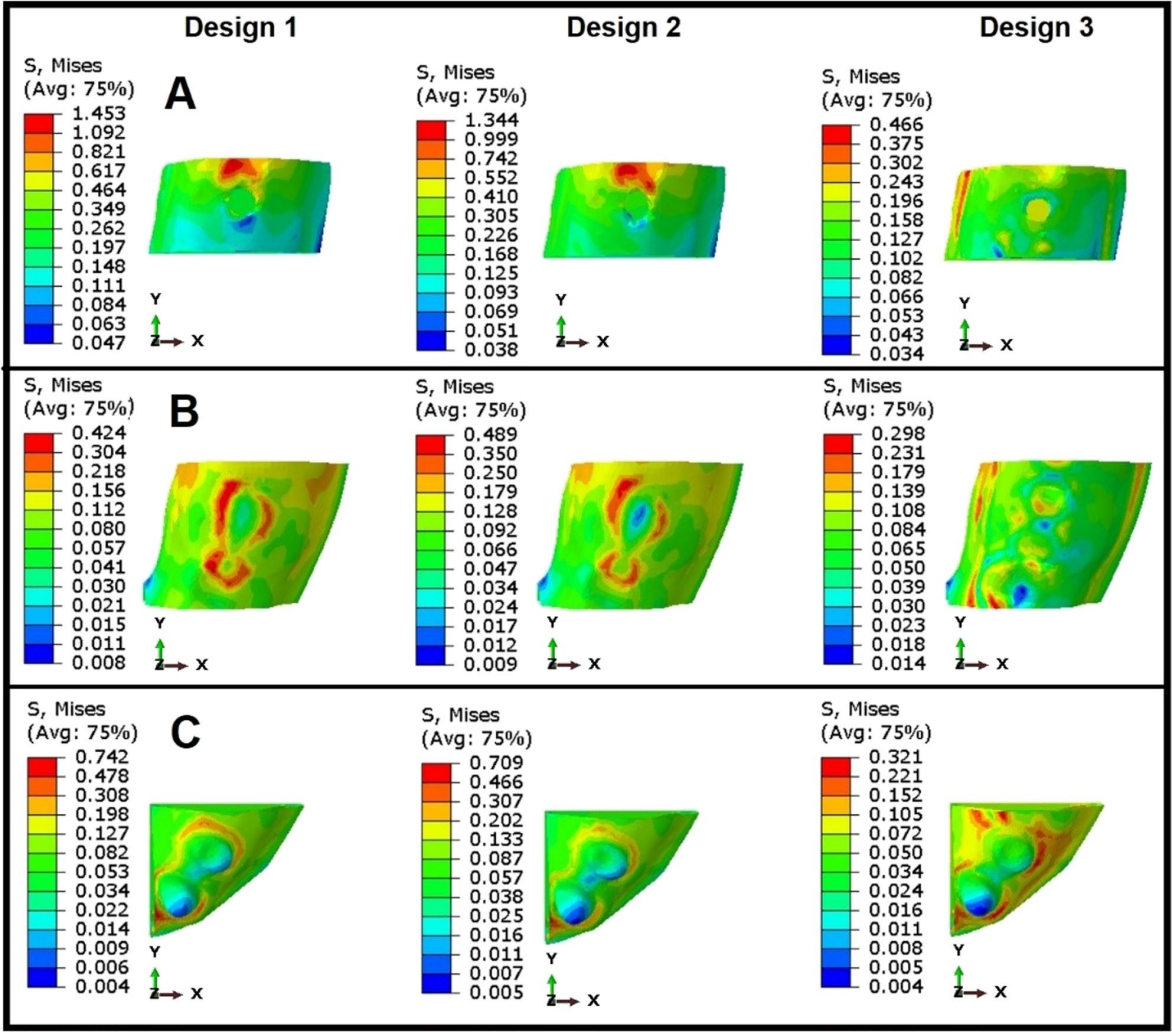
The von Mises stress levels induced in the three designs (occlusal view), (**A**) around the implants, (**B**) the edentulous ridge, and (**C**) around the abutments

**Fig. 4:**
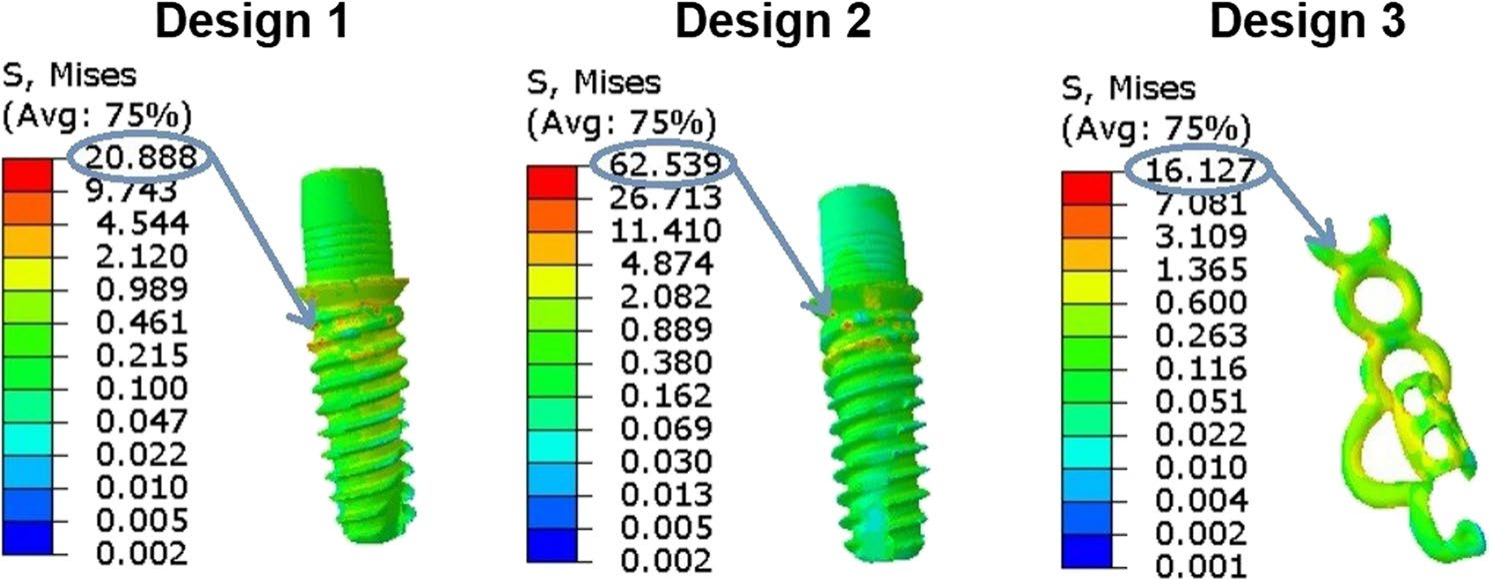
The maximum von Mises stress found for each design

The von Mises stress levels induced around the abutments (the canine, 1st premolar), over the edentulous ridge, and around the implant are recorded and compared across all designs, as illustrated in (Fig. 3). Additionally, the maximum von Mises stress for each design is recorded, as shown in (Fig. 4; Table 2).

**Table 2:**
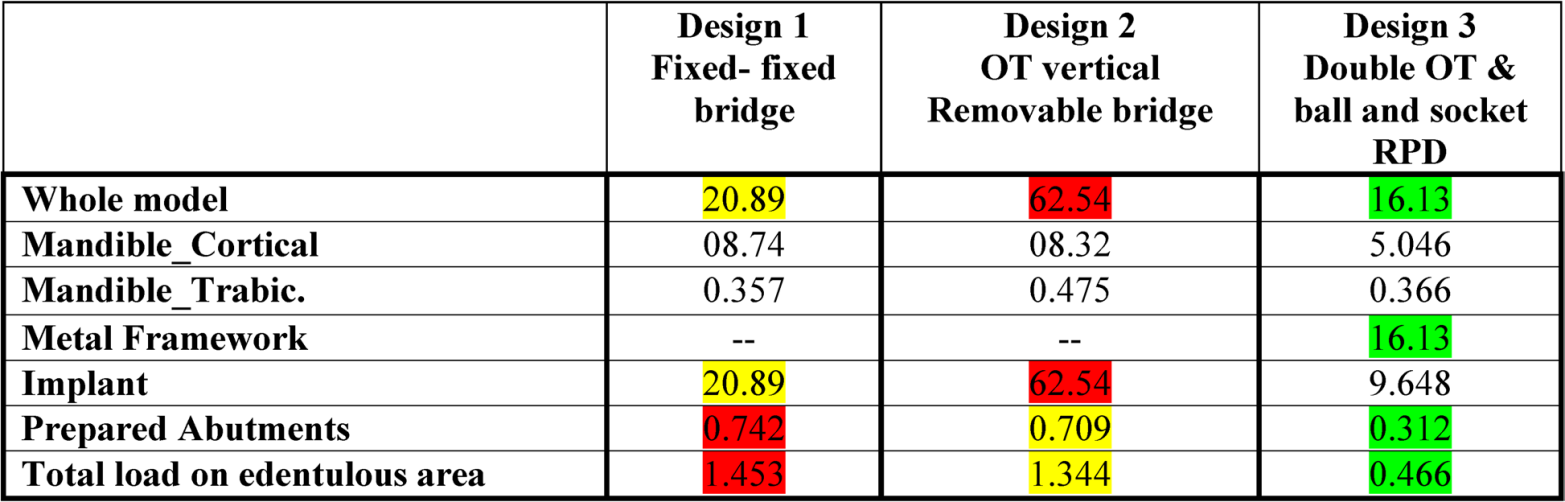
Maximum von Mises stresses in the three designs measured in (MPa)

Red color indicates the maximum stresses found in the three designs, green color indicates the lowest stresses found in the three designs, while the yellow color indicates the moderate stresses found in the three designs

**Fig. 5:**
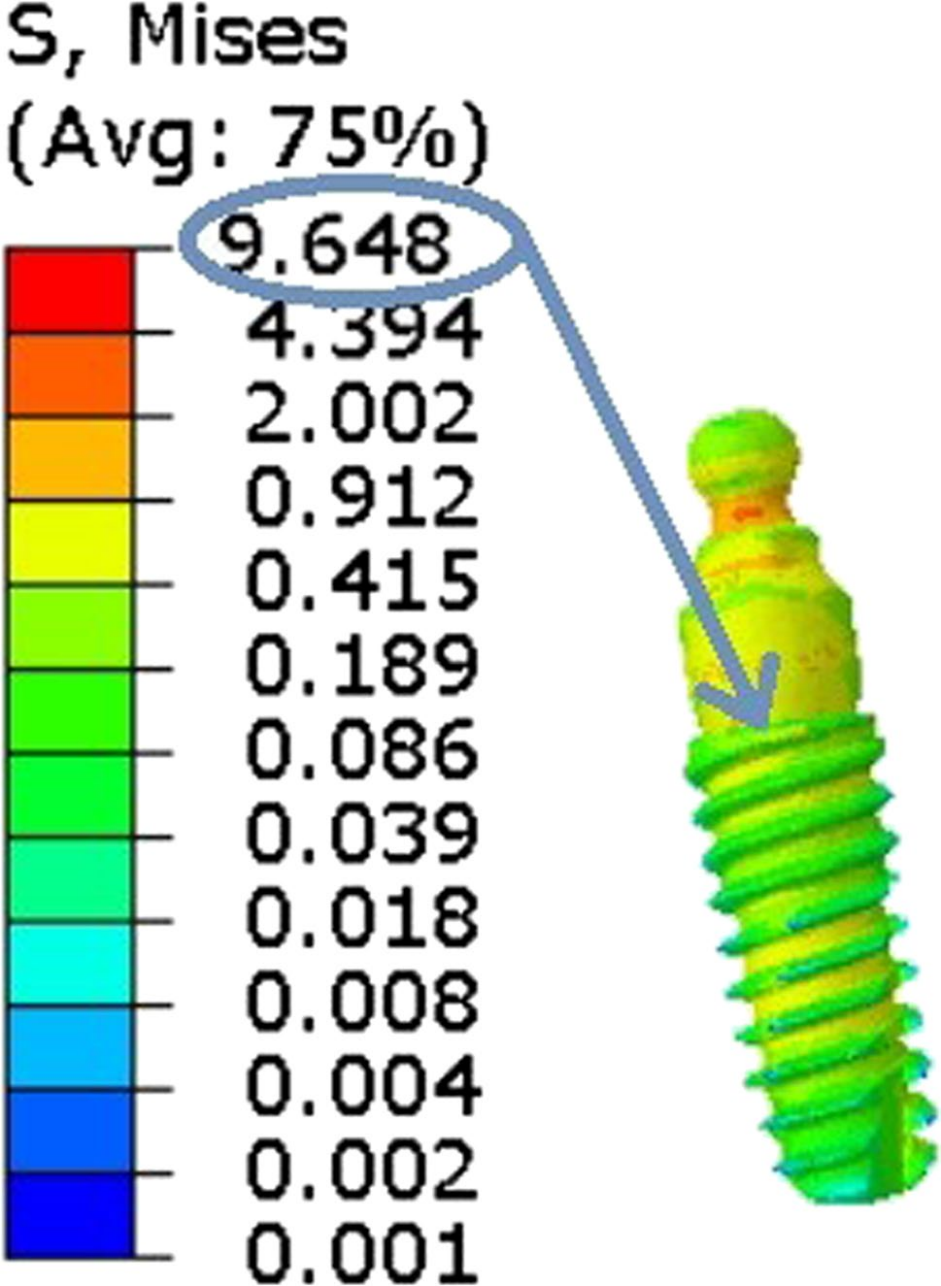
Design 3 showed the least von Mises stresses around the implant

Regarding the results of this study, the lowest von Mises stresses were recorded in design 3 for the abutments, the edentulous ridge, and the implant (Fig. 5). However, the highest von Mises stresses were recorded around the abutments and over the edentulous ridge in design 1, finally, the highest von Mises stresses were recorded around the implant in design 2.

## Discussion

Restoration of free-end saddle cases is always considered a challenging situation due to the absence of support at the posterior end of the saddle. This was addressed in this study by placing a posterior implant at the second molar area, which provided adequate retention and support, allowing the patient to perform mastication with comfort and efficiency [35].

Uludag and Celik [36] had stated the advantages of unilateral design PD, including the ease of use and the absence of the major connector; however, the possibility of dislodgement remains a serious problem that may lead to more complications like denture aspiration or swallowing; therefore, they offered the use of a single distal implant that modifies Kennedy class II to Kennedy class III acting as a distal abutment that provides more retention, stability, and support.

Digital designing of different treatment options in this study aimed to optimize the RPD design through computational methods using the standardized virtual dental arches, it was demonstrated that virtual reconstruction of numerous cases was feasible with great improvement in denture adaptation to the underlying mucosa, minimizing the related clinical complications and enhancing the clinical outcome [37, 38].

For better aesthetics, metal crowns with porcelain-facing were used. To ensure better load distribution across multiple teeth, splinting of the abutment teeth (canine and first premolar) was done to improve support and, as a result, improve the dental prosthesis’s prognosis [39].

It is well known that the functional behavior of the abutment teeth and the denture base is significantly influenced by the precise selection of direct retainers [40]. In this study, three retainers with different connection rigidity were selected. design 1, the fixed-fixed rigid connection bridge, followed by design 2, the OT vertical attachment that allows vertical resiliency, and finally design 3, the double OT attachment anteriorly with ball & socket attachment posteriorly allows resiliency in all directions.

For design 1, choosing a fixed-fixed bridge offers the advantages of a prosthesis that has no flanges, with its small size, not crossing the contralateral side, in addition to better neuromuscular coordination, and maintenance of proprioception for the patients.

For design 2, choosing the OT vertical attachment that allows vertical resiliency between the removable bridge and the abutment is thought to reduce stress exerted on the supporting structures. At the implant side, the OT vertical attachment was oriented to be used as an intra-coronal attachment to get the benefits of improved esthetics and decreased food stagnation (avoiding the use of metal clasps), with more apical direction force application to the implant compared with occlusal rest, reducing the lever arm and minimizing the torquing forces. Additionally, a more favorable force direction was also obtained as close as possible to the long axis of the implant. However, it requires adequate facio-lingual width/cervico-occlusal height to promote adequate friction between the attachment parts, which may lead to extensive abutment preparation ending with root canal treatment to obtain more room for this keyway retention mechanism type. Accordingly, the OT vertical attachment at the abutment side was oriented to be used as an extra-coronal attachment to connect the fixed bridge with the anterior abutment teeth, as it does not modify the normal abutment contours. The attachment is placed entirely outside the tooth contour, offering an easy selection of a suitable path of insertion of the PD, with great freedom in its component design. It also allows for abutment splinting for wider force distribution. It does not require a great space regarding abutment’s width and height; hence, it can be used when intraoral attachment is contraindicated due to space limitations [3, 41].

For design 3, choosing a double OT cap attachment anteriorly and ball &socket attachment posteriorly that offers the advantage of resiliency of the removable denture in all directions.

When the three different treatment modalities were constructed and compared regarding stresses transmitted to the supporting abutments anteriorly, the edentulous ridge, and around the implant posteriorly it was found that, the stresses induced within the whole design, and within the implant were found to be the maximum in design 2 (OT vertical attachments retained removable bridge) and followed by design 1 (fixed-fixed bridge). The stresses induced over the edentulous ridge, and the prepared abutments, were observed to be the maximum in design 1 (fixed- fixed bridge) followed by design 2 (OT vertical attachments retained removable bridge), while in design 3 (double OT attachment retained unilateral RPD) had induced the minimum stresses to the anterior abutments, the edentulous ridge, and around the posterior implant as shown in (Fig. 5).

Regarding design 3, a double OT cap was utilized anteriorly, while a ball and socket attachment was utilized posteriorly. Ball attachments allow movement of the denture in all directions. The unique resilient design of the OT unilateral attachment -the design of two spheres in different planes- promotes load distribution in a more favorable direction during mastication, which cancels the need for cross-arch stabilization and unnecessary components like the major connector. Besides, the enhanced retention obtained, the better tolerance, and the greater denture adaptation. The combination of using a resilient double OT attachment with a ball and socket attachment on the implant distal abutment also allows more load distribution [42]. In resilient-resilient attachment, the stress distribution pattern was found to be the most favorable, and denture support could be obtained from the residual edentulous ridge, preventing the overload to implants and abutments [43].

Regarding design 2, (OT vertical attachment retained removable bridge that allows resiliency in the vertical direction). Stresses were recorded to be lower than those recorded in design 1 on the anterior natural abutments and on the edentulous ridge. The resilient effect, together with the centralization of the attachment over the ridge buccolingually, allows better load distribution and preservation of the ridge and abutment health [44, 45].

While the stresses exerted on the implant side when the attachment was oriented to be intra-coronal, were found to be higher than design 1, this may be explained by a previous study compared the stress distribution in tooth -implant supported prosthesis with resilient- resilient connection in one group and rigid- rigid connection in the other group, the author had observed that non-rigid attachment doesn’t carry the excessive load to the underlying edentulous ridge otherwise it concentrate the stresses inside the attachment body and consequently transmit the stress to the implant, regarding this situation, the support gained from the natural abutments was less, and as a result, a substantial cantilever is formed on the implant (this may explain the results of the current study). Moreover, attachment’s component wear and implant failure in the rigid-rigid connection group are found to be increased compared to the resilient-resilient connection group, adding that the implant does not have periodontal ligament, so cushion effect is not present [46].

It was reported that the morphology of the implant attachment influences prosthesis movement and stress distribution, especially in the cortical bone around the implant neck region. Ohyama et al. [47], concluded that prosthesis movement is more limited when high attachments (greater than 2 mm) are used, this goes with the results of this study as the OT vertical attachment height was about 4 mm, so it permits limited movement compare to design 3, and also explain the near values of stresses exerted on the abutments (0.742 − 0.709) and on the residual ridge (1.453–1.344 ) recorded in design 1 and design 2 respectively.

It was observed that rigid retainers, such as rigid precision attachments more efficiently reduce denture base displacement compared to non-rigid connections. This was explained by the fact that flexible connection attachments have stress-releasing action that tends to dissipate the stress exerted on the abutment teeth towards the denture base, while rigid connection attachments concentrate the stresses on the abutments. An in vitro study was done to investigate abutment mobility resulting from stresses exerted by different distal extension RPDs, and it was proved that the load that was exerted on the denture base retained by high-rigidity direct retainers was found to be smaller than that of the lower-rigidity direct retainers [36]. The same findings were observed in this study. On the other hand, the effect of the retainer’s degree of rigidity on abutments and denture base displacement was studied by Itoh et al. [48] They claimed that no significant difference was found regarding denture base and abutment teeth displacement in the apical direction with direct retainers’ different degrees of rigidity.

Regarding design I, (non-resilient connection) exerted the highest stresses on the supporting abutments and the residual ridge. This may be explained as this type of prosthesis doesn’t allow any type of movement between its components, either vertical or rotational, it does not have a shock-absorbing component. Consequently, all the stresses are mainly directed to and concentrated on the supporting tissues [49].

### Regarding the connection of natural teeth with an implant

It is essential to recognize that the decision to connect a natural tooth to an implant remains controversial in prosthodontics due to the inherent biomechanical differences between teeth (which exhibit physiologic mobility via the periodontal ligament) and implants (which are ankylotically rigid) [6, 50]. However, when specific guidelines are followed—including the use of appropriate connector designs and careful case selection—tooth-implant supported prostheses demonstrate acceptable survival rates ranging from 91.8% to 100% over observation periods of 6 to 180 months [51]. The present study evaluates three distinct connector configurations within this treatment category, each offering a different biomechanical profile for stress distribution to the supporting structures.

### Design 1: fixed-fixed bridge

The fixed-fixed tooth-implant supported bridge is suggested to be indicated when the anterior abutment teeth demonstrate sufficient periodontal health and structural integrity with minimal mobility (Class 0 or I according to Miller’s classification) to accept a share of the occlusal load, and when the edentulous ridge is deemed capable of tolerating the stresses transmitted through the prosthesis, and the patient has no preference for prosthesis removability [52, 53]. In this configuration, the rigid connection between the anterior abutments and the posterior implant results in maximum stress transmission to the natural teeth and the ridge, as observed in the present study.

### Design 2: OT vertical attachment-retained removable bridge

The OT vertical attachment-retained removable bridge transfers maximum stress to the posterior implant while reducing stress on the anterior abutments and the edentulous ridge compared to the fixed-fixed configuration. It is suggested to be indicated when protection of the anterior abutment teeth is a primary clinical concern (periodontally compromised (Class I or II mobility), and a degree of prosthesis removability is desirable for hygiene access. (a), also, when preservation of remaining natural tooth support is prioritized over implant protection, and the posterior implant is of adequate dimensions and bone support to tolerate higher stress concentrations, and finally, when the patient or clinician desires retrievability for maintenance of peri-implant and periodontal health [54, 55].

### Design 3: double OT attachment-retained unilateral RPD

The double OT attachment-retained unilateral removable partial denture is suggested to be indicated when minimal stress transmission to all supporting structures—anterior abutments, edentulous ridge, and posterior implant—is the primary therapeutic objective. The present study observed that this design induced the lowest stresses among the three configurations to the anterior abutments, the edentulous ridge, and the peri-implant bone. Therefore, it is most appropriate when ( the anterior abutment teeth, the edentulous ridge, and the posterior implant are considered biomechanically vulnerable (e.g., short or narrow implant, reduced bone density with significant resorption, periodontally compromised anterior teeth), a conservative biomechanical approach is desired, and the patient accepts a unilateral removable prosthesis design. The double OT attachment system provides a resilient connection that dissipates occlusal forces across a broader area, thereby reducing peak stresses on any single supporting structure. This design is particularly valuable in elderly patients or those with systemic conditions that impair bone healing or increase susceptibility to biomechanical overload [56].

### Biomechanical validity of the model

- This study used a controlled computational framework to compare the biomechanical performance of different design configurations under consistent conditions. While effective for identifying relative performance trends, the approach lacks physical validation, meaning results should be seen as indicators of relative behavior rather than absolute physiological representations. This study provides a foundation to guide future experimental validations and to translate computational insights into clinically meaningful outcomes. Additionally, the deterministic nature of the finite element analysis (FEA) means that fixed inputs produce singular outcomes (stress, strain, deformation), so conventional statistical methods for quantifying uncertainty do not apply. Instead, the findings are validated through mesh convergence analysis and adherence to continuum mechanics principles, ensuring computational integrity.
- The decision not to model the periodontal ligament (PDL) in the finite element analysis was deliberate and justified for several reasons: the PDL’s complex nonlinear behavior is difficult to simulate accurately; including it would increase computational demands and cause convergence issues; and prior research shows that while the PDL affects absolute stress values, it does not significantly alter relative comparative trends between designs when identical simplified conditions are applied. Since the study’s primary goal was to compare relative stress patterns among three treatment modalities under the same boundary conditions, excluding the PDL preserves internal validity. All groups were modeled equally, so any influence of the simplification applies uniformly across all, ensuring the comparative analysis remains reliable [57–61]. It explicitly acknowledges the well-documented difficulties in fully replicating PDL anatomy and mechanics in conventional FEA. We also clarify that our simplified model represents a conservative, worst-case condition and that, given the comparative nature of our study, the relative stress patterns among prosthetic designs remain valid [61–65].

### Limitations of the study

1. Only FEA was used to assess the intricate pattern of stress encountered by implants and abutments. So, further clinical validation is required.
2. The loading force did not take into account various opposing dentition scenarios; instead, it reflected an average biting force for the implant-supported PD.
3. Simulating the biphasic (solid-fluid) nature of the PDL under static load would require transient fluid-structure interaction analysis, which was beyond the scope of this comparative study.
4. Future studies incorporating a nonlinear PDL are needed to confirm the absolute stress values, but the comparative trends between connection designs are likely preserved given the model’s conservative boundary conditions.

## Conclusions

Within the limitations of this study, it could be concluded that:

1. The double OT retained unilateral PD offered the lowest stresses applied on the abutment, the residual ridge, and the implant.
2. The intra-coronal attachment side of the OT vertical attachment design used to retain the implant was found to apply more stress on the implant compared to the other two designs.
3. The fixed-fixed bridge design exerted higher stresses on the abutment side and on the residual ridge, compared to the extra-coronal attachment. Connecting teeth with an implant may be considered as a successful treatment option when a separate, independent support between teeth and implant is needed, including the use of minimally mobile abutments with a favorable crown/root ratio and successful implant osseo-integration. However, clinical recommendations cannot be drawn definitively from the current deterministic FEA model alone.
4. The absence of the PDL provides a ‘stiffer’ support condition, representing a worst-case scenario for stress concentration at the natural tooth abutments. Designs that perform well under these rigid conditions are expected to perform at least as well clinically.

## Data Availability

NO data availability; there are no data underlying the manuscript; all data were presented by figures and tables at the current study.

## Abbreviations

FEA: Finite element analysis
PD: Partial denture
RPDs: Removable partial dentures
FPDs: Fixed partial dentures
PFM: Porcelain fused to metal
TISP: Tooth-implant supported prosthesis
STL: Standard Tessellation Language
